# High-dose postpartum thromboprophylaxis in women at high risk of pregnancy-related venous thromboembolism: a single-center prospective cohort study

**DOI:** 10.1016/j.rpth.2025.102846

**Published:** 2025-04-04

**Authors:** Sean C.S. Rivrud, Èmese R.H. Heijkoop, Marloes A.G. Holswilder–Olde Scholtenhuis, Karina Meijer

**Affiliations:** 1 Department of Intensive Care, University Medical Center Groningen, Groningen, The Netherlands; 2 Department of Hematology, University Medical Center Groningen, Groningen, The Netherlands; 3 Department of Obstetrics and Gynecology, University Medical Center Groningen, Groningen, The Netherlands

**Keywords:** anticoagulants, heparin, low-molecular-weight, pregnancy, thrombophilia, thrombosis

## Abstract

**Background:**

Pregnancy-related venous thromboembolism (VTE) is a major cause of maternal mortality and morbidity. While thromboprophylaxis can reduce the incidence of VTE, it may increase the risk of bleeding. Current guidelines recommend assessing VTE risk in pregnant women and administering low-molecular-weight heparin (LMWH) thromboprophylaxis to those at high risk. However, there is a paucity of evidence regarding the optimal dosing of postpartum LMWH thromboprophylaxis.

**Objectives:**

To evaluate the safety and efficacy of fixed low-dose LMWH antepartum and weight-based high-dose LMWH (equivalent to weight-based therapeutic-dose LMWH) until 6 weeks postpartum in a prospective cohort of women at high risk for pregnancy-related VTE.

**Methods:**

From December 8, 2014, to November 9, 2023, we included patients at high risk for pregnancy-related VTE who required thromboprophylaxis during pregnancy and the puerperium. The primary safety outcome was the incidence of primary and secondary major postpartum hemorrhage. The secondary safety outcome was the incidence of primary and secondary postpartum clinically relevant nonmajor bleeding (CRNMB). The efficacy outcome was the incidence of VTE. Additional outcomes included treatment discontinuation and treatment modification.

**Results:**

We found a 6.56% incidence of primary major postpartum hemorrhage, a 9.84% incidence of primary postpartum CRNMB, a 5.00% incidence of secondary postpartum CRNMB, a 3.33% incidence of VTE, a 16.1% incidence of treatment discontinuation, and a 30.6% incidence of treatment modification.

**Conclusion:**

When evaluating the optimal dose of thromboprophylaxis, the acceptable residual risk of VTE is debatable but should be considered in conjunction with the risks of adverse events, particularly bleeding and drug reactions, such as hypersensitivity skin reactions.

## Essentials

• Pregnancy-related VTE is a leading cause of maternal mortality and morbidity.
• High-risk patients received low-dose LMWH antepartum and high-dose LMWH postpartum.
• VTE occurred in 3.33% of patients; major postpartum hemorrhage (PPH) occurred in 6.56%.
• Balancing VTE prevention and bleeding risk is crucial for postpartum thromboprophylaxis.

## 1 Introduction

Hemostatic and hemodynamic changes, including increased procoagulant activity, a decrease in physiological anticoagulants, and progressive venous stasis in lower limbs, occur throughout pregnancy and the puerperium [[1], [2], [3], [4]]. Consequently, women are at a particularly high risk of venous thromboembolism (VTE) in this period [5]. In turn, pregnancy-related VTE, encompassing pulmonary embolism (PE), or deep vein thrombosis (DVT), is a major cause of maternal mortality and morbidity [5]. In pregnant women at high risk of VTE, thromboprophylaxis during pregnancy and the puerperium may reduce the incidence of VTE but also increase the risk of bleeding [6]. Additionally, adverse events, such as hypersensitivity skin reactions, injection-related pain, and bruising at the injection site, are common when using thromboprophylaxis—especially over a prolonged period [7,8]. Therefore, careful consideration of fetal and maternal outcomes is pertinent when considering the use of thromboprophylaxis in pregnancy. To confer the most favorable balance between harms and benefits, guidelines recommend assessing the risk of pregnancy-related VTE in all pregnant women and administering thromboprophylaxis to women who are deemed to be high risk [6,[9], [10], [11], [12]].

Low-molecular-weight heparin (LMWH) is the anticoagulant class of choice for thromboprophylaxis during pregnancy and in the puerperium due to its safety profile, and current guidelines recommend low or intermediate doses [6,[9], [10], [11], [12]]. Some have advocated for thromboprophylaxis with higher doses of LMWH due to the observed rates of thromboprophylaxis failure when using fixed low-dose LMWH [13,14]. Nonetheless, the theoretical benefits of higher doses may be offset by an increase in adverse events. Indeed, the recently conducted Highlow study concluded that fixed low-dose LMWH is the appropriate dose for antepartum thromboprophylaxis [15]. However, the study could not ascertain a clear preference in the puerperium, as the primary analysis showed no significant difference between dosing strategies. Post hoc analysis suggested that intermediate-dose LMWH may be more effective, with a lower risk of PE (relative risk, 0.11 [95% CI, 0.01-0.87]) and VTE or superficial thrombophlebitis up to 6 weeks postpartum (relative risk, 0.45 [95% CI, 0.24-0.85]) [15]. As most pregnancy-related VTEs in high-risk patients occur in this period [15,16], more evidence on the safety and efficacy of higher doses of LMWH in the puerperium is crucial to inform future guidelines and mitigate the burden of pregnancy-related VTE.

Our hospital protocol states that women at high risk of pregnancy-related VTE should receive fixed low-dose LMWH antepartum and weight-based high-dose LMWH (equivalent to therapeutic-dose LMWH based on prepregnancy weight) after delivery until 6 weeks postpartum (referred to here as the puerperium). In the current study, we describe a prospective cohort of patients who received thromboprophylaxis according to this protocol. We aimed to assess the safety of said protocol by the incidence of postpartum hemorrhage (PPH) and postpartum clinically relevant nonmajor bleeding (CRNMB) and its efficacy by the incidence of VTE.

## 2 Methods

The current study is a prospective cohort study designed to evaluate the safety and efficacy of fixed low-dose LMWH antepartum and weight-based high-dose LMWH (equivalent to therapeutic-dose LMWH based on prepregnancy weight) in the puerperium in pregnant women at high risk for VTE. This study is reported according to the Strengthening the Reporting of Observational Studies in Epidemiology guideline for cohort studies (Supplementary Table S1) [17].

### 2.1 Study design and participants

We prospectively and consecutively included patients at high risk for pregnancy-related VTE at the University Medical Center of Groningen between December 8, 2014, and November 9, 2023. We included all patients with an established indication for thromboprophylaxis during pregnancy and the puerperium, either determined after referral to the Department of Hematology or in accordance with our local hospital protocol shortly prior to referral. Indications for thromboprophylaxis during pregnancy and puerperium, according to our local protocol, include (1) a single episode of VTE provoked by the use of oral contraceptives, pregnancy, the postpartum period, or without a clear provoking factor (regardless of the presence of thrombophilia), (2) a history of recurrent VTE, and (3) homozygous factor (F)V Leiden, homozygous prothrombin mutation, or antithrombin, protein C, or protein S deficiency with a family history of VTE. In high-risk cases where a paucity of evidence precluded the formulation of definitive recommendations in the Dutch national guidelines—such as for obstetric antiphospholipid syndrome—the decision to initiate thromboprophylaxis according to the protocol was left to the discretion of the attending hematologist [18]. Patients with an indication for therapeutic-dose LMWH prepregnancy were not considered for the current study.

All patients received fixed low-dose LMWH antepartum and weight-based high-dose anticoagulation in the puerperium. In the current study, 2850 anti-Xa International Units (IU) of nadroparin, 4000 anti-Xa IU of enoxaparin, 5000 anti-Xa IU of dalteparin, and 2.5 mg of fondaparinux administered once daily were considered fixed, low doses of thromboprophylaxis. The weight-based high doses were based on prepregnancy weight and were identical to weight-based therapeutic doses. The exact doses per weight category can be appreciated in detail in Supplementary Table S2. The preferred anticoagulant at our institution is nadroparin. In cases where nadroparin was poorly tolerated, enoxaparin, dalteparin, or tinzaparin (only postpartum since prophylactic doses of tinzaparin were not available) were administered. Treatment with fondaparinux was reserved for patients who were intolerant to all aforementioned LMWHs.

Regarding peripartum management of thromboprophylaxis, we advised discontinuation of antepartum thromboprophylaxis 24 hours before a planned induction of labor or planned cesarean section. If no such intervention was planned, the patient was instructed to discontinue thromboprophylaxis upon experiencing contractions or membrane rupture. Finally, we advised initiation of postpartum thromboprophylaxis at least 6 hours after delivery, provided hemostasis had been achieved, as determined by the attending physician.

All data were recorded by a physician using a standardized survey directly following patient consults. Missing or unknown data were retrieved from electronic patient records when possible. We followed all patients until 6 weeks postpartum and concluded the follow-up with a (telephone) consult. In cases where we had treated a woman throughout multiple pregnancies, we only included the first pregnancy to avoid biases, such as selection bias.

Consent was obtained from all participants. According to national legislation and the University Medical Center of Groningen’s ethical committee, the current study is exempt from protocol review as it is purely observational with no experimental intervention.

### 2.2 Outcomes

The primary safety outcome was the incidence of major PPH according to the population-specific International Society on Thrombosis and Haemostasis (ISTH) criteria [19]. Primary major PPH was defined as PPH within 24 hours of delivery that necessitates transfusion of 2 or more units of whole blood or red cells to maintain hemoglobin levels higher than 7 to 9 g/dL. Additionally, it includes cases with blood loss exceeding 1000 mL within 24 hours of delivery that require urgent intervention to stop bleeding, secondline treatment with uterotonics, balloon tamponade, embolization, conservative surgery, or hysterectomy. It also includes cases resulting in death within 24 hours of delivery following a blood loss of more than 1000 mL. Secondary major PPH was defined as acute clinically overt bleeding between 24 hours and 6 weeks postpartum that leads to death, occurs in a critical organ, is associated with a fall in hemoglobin greater than 2 g/dL, or necessitates transfusion of 2 or more units of whole blood or red cells to maintain hemoglobin levels higher than 7 to 9 g/dL [19].

The secondary safety outcome was the incidence of postpartum CRNMB, as per the population-specific ISTH criteria [19]. Primary postpartum CRNMB was defined as blood loss of less than 1000 mL requiring uterine intervention, first-line treatment with uterotonics and/or tranexamic acid, or blood loss exceeding 1000 mL necessitating first-line treatment with uterotonics and/or tranexamic acid, all occurring within 24 hours postpartum. Secondary postpartum CRNMB was defined as vaginal bleeding requiring hospitalization, modification of thromboprophylaxis therapy, or any sign or symptom of bleeding prompting face-to-face evaluation and necessitating hospitalization or medical intervention, occurring between 24 hours and 6 weeks postpartum.

The efficacy outcome was the incidence of VTE, defined as objectively proven DVT or PE diagnosed by compression ultrasonography, computed tomographic angiography, or magnetic resonance imaging.

Additional outcomes of interest were treatment discontinuation and treatment modification under the current protocol. Treatment discontinuation was defined as the premature cessation of treatment according to our protocol, and treatment modification was defined as a switch to another anticoagulant preparation. Treatment compliance was self-reported.

### 2.3 Statistical analysis

All outcomes were presented as percentages with 95% CIs calculated according to the Clopper–Pearson exact method. The distribution of continuous variables was evaluated using density plots and assessed using the Shapiro–Wilk normality test. When the distribution of a variable was normal, it was described in terms of its mean and SD. Otherwise, it was described in terms of its median and IQR. Post hoc comparisons of dichotomous outcomes were made employing the chi-squared test, the difference in proportions, and the 95% CIs of the difference in proportions. For studies in which we aimed to compare incidences but lacked reported CIs, we conducted a post hoc analysis of their published data and calculated the CIs using the Clopper–Pearson exact method. All analyses were performed using R software (R Core Team, 2023, version 4.3.2).

## 3 Results

We included a total of 62 patients at high risk for pregnancy-related VTE who had an indication for fixed low-dose LMWH antepartum and weight-based high-dose LMWH in the puerperium, according to our hospital protocol. The mean age was 33.0 years (SD, 5.20), and the median weight before pregnancy was 73.0 kg (IQR, 62.0-86.0). The indication for thromboprophylaxis in the majority of the cohort was a personal history of VTE (*n* = 46/62 [74.2%]) provoked by the use of the oral contraceptive pill (*n* = 26/46 [56.5%]) or a previous pregnancy (*n* = 12/46 [26.1%]). In 8 of the patients with a personal history of VTE, there was no clear provoking factor (*n* = 8/46 [17.4%]. Of the patients receiving thromboprophylaxis due to a personal history of VTE, 1 was a FV Leiden heterozygote (*n* = 1/46 [2.17%]), and the rest were not known to have thrombophilia (*n* = 45/46 [97.8%]). Eight patients were considered at high risk for VTE during pregnancy due to thrombophilia and a family history of VTE (*n* = 8/62 [12.9%]), of which 5 had a protein C deficiency (*n* = 5/8 [62.5%]), 1 had an antithrombin III deficiency (*n* = 1/8 [12.5%]), 1 had a protein S deficiency (*n* = 1/8 [12.5%]), and 1 had a FV Leiden mutation (*n* = 1/8 [12.5%]). The remaining 8 had other indications for anticoagulation, including obstetric antiphospholipid syndrome (*n* = 4/62 [6.45%]), a personal history of cerebral venous sinus thrombosis (*n* = 3/62 [4.84%]), and Klippel–Trénaunay syndrome (*n* = 1/62 [1.61%]). The mode of delivery was principally vaginal (*n* = 52/62 [83.9%]). Nine patients underwent cesarean delivery (*n* = 9/62 [14.5%]), and pregnancy resulted in a miscarriage in 1 patient (*n* = 1/62 [1.61%]). The patient characteristics and outcomes are listed in Tables 1 and 2, respectively.

**Table 1:** Patient characteristics.

Variable	*N* = 62^a^
Age at delivery (y)	33.0 (5.20)
Weight before pregnancy (kg)	73 (62.0, 86.0)
Primigravidity	20/62 (32.3)
Nulliparity	26/62 (41.9)
Gestational age at initiation of thromboprophylaxis (wk)	6 (5, 8)
*Indication of anticoagulation*	
Personal history of VTE	46/62 (74.2)
Known thrombophilia and family history of VTE	8/62 (12.9)
Other	8/62 (12.9)
*VTE provoking factor*	
Related to the use of oral contraceptives	26/46 (56.5)
Pregnancy-related	12/46 (26.1)
None	8/46 (17.4)
*Mode of delivery*	
Vaginal delivery	52/62 (83.9)
Primary cesarean section	3/62 (4.84)
Secondary cesarean section	6/62 (9.68)
Miscarriage	1/62 (1.61)

VTE, venous thromboembolism.

a Mean (SD); median (IQR); n/N (%)

**Table 2:** Outcomes.

Outcome	Incidence^a^	95% CI^b^
*Primary safety outcomes*		
Primary major PPH	6.56 (4/61)	1.82-15.9
Secondary major PPH	0.00 (0/60)	0.00-5.96
*Secondary safety outcomes*		
Primary postpartum CRNMB	9.84 (6/61)	3.70-20.2
Secondary postpartum CRNMB	5.00 (3/60)	1.04-13.9
*Efficacy outcomes*		
Antepartum VTE	0.00 (0/60)	0.00-5.78
Postpartum VTE	3.33 (2/60)	0.406-11.5
*Additional outcomes*		
Treatment discontinuation	16.1 (10/62)	8.02-27.7
Treatment modification	30.6 (19/62)	19.6-43.7

CRNMB, clinically relevant nonmajor bleeding; PPH, postpartum hemorrhage; VTE, venous thromboembolism.

a % (n/N).

b Lower CI (%)-upper CI (%).

### 3.1 Incidence of major PPH

Four patients experienced primary major PPH (*n* = 4/61; 6.56% [95% CI, 1.82%-15.9%]). One patient developed disseminated intravascular coagulation with massive blood loss and hemodynamic shock following a total placental abruption and sequential intrauterine fetal demise, necessitating an emergency (secondary) cesarean section admission to the intensive care unit (*n* = 1/4 [25.0%]). This patient had stopped fixed low-dose LMWH more than 24 hours prior to surgery and resumed the fixed low-dose regimen the day after delivery until 6 weeks postpartum and thus did not complete treatment according to our hospital protocol. Two patients experienced significant blood loss due to retained placenta following vaginal delivery, requiring manual extraction (*n* = 2/4 [50.0%]). One of these 2 patients had stopped low-dose LMWH 12 to 24 hours prior and the other more than 24 hours prior, and both received high-dose LMWH within 24 hours postpartum. The fourth patient experienced extensive vaginal, bilateral labial, and perineal ruptures, including injury to the anal sphincter during vaginal delivery, resulting in a drop in hemoglobin concentration to 6.8 g/dL and requiring transfusion of 3 units of red blood cells (*n* = 1/4 [25.0%]). Low-dose LMWH had been stopped more than 24 hours before delivery, and high-dose LMWH was administered within 24 hours postpartum. None of the patients who experienced primary major PPH had received high-dose LMWH prior to the bleeding event. Secondary major PPH was not recorded in any patients (*n* = 0/60; 0.00% [95% CI, 0.00%-5.96%]).

### 3.2 Incidence of postpartum CRNMB

Primary postpartum CRNMB was reported in 6 patients (*n* = 6/61; 9.84% [95% CI, 3.70%-20.2%]). Most of the patients that experienced CRNMB had vaginal deliveries resulting in significant blood loss, requiring first-line uterotonics (*n* = 5/6 [83.3%]). One underwent a cesarean section, resulting in significant blood loss, necessitating administration of tranexamic acid (*n* = 1/6 [16.7%]). Low-dose LMWH was discontinued more than 24 hours before delivery in half of the cases (*n* = 3/6 [50.0%]), between 12 and 24 hours before delivery in 2 cases (*n* = 2/6 [33.3%]), and less than 12 hours prior in 1 case (*n* = 1/6 [16.7%]). High-dose LMWH was administered within 24 hours postpartum in all cases, with no patients receiving high-dose LMWH before the bleeding events.

Secondary postpartum CRNMB was reported in 3 patients (*n* = 3/60; 5.00% [95% CI, 1.04%-13.9%]). The median onset of secondary CRNMB was 19.0 days postpartum (IQR, 15.5-20.5). One patient discontinued high-dose LMWH after 22 days due to persistent bleeding at injection sites and associated pain (*n* = 1/3 [33.3%]). One patient was hospitalized 19 days postpartum for vaginal blood loss, which prompted the cessation of high-dose LMWH for the remainder of the puerperium (*n* = 1/3 [33.3%]). The third patient presented with vaginal bleeding 12 days postpartum, resulting in a discontinuation of high-dose LMWH (*n* = 1/3 [33.3%]).

### 3.3 Incidence of VTE

Postpartum VTE was diagnosed in 2 patients (*n* = 2/60; 3.33% [95% CI, 0.406%-11.5%]). Both events were DVTs. One DVT occurred 21 days postpartum in a patient with a personal history of VTE provoked by the use of the oral contraceptive pill. This DVT was isolated to the deep and common femoral veins of the right lower limb (*n* = 1/2 [50.0%]). The other DVT occurred in a patient with antithrombin III deficiency with a positive family history of VTE. In this patient, the DVT occurred 11 days postpartum and extended from the popliteal veins into the external iliac vein of the left lower limb (*n* = 1/2 [50.0%]). Both DVTs occurred while the patients were receiving high-dose LMWH. No VTEs were reported antepartum (*n* = 0/62; 0.00% [95% CI, 0.00%-5.78%]).

### 3.4 Treatment discontinuation and treatment modification

In 10 patients, treatment according to our protocol was discontinued prematurely (*n* = 10/62; 16.1% [95% CI, 8.02%-27.7%]). In 5 of the 10 cases (50.0%), this was due to patient noncompliance, motivated by nadroparin-induced hypersensitivity skin reactions (*n* = 1/5 [20.0%]), pain or discomfort (*n* = 2/5 [40.0%]), or unspecified reasons (*n* = 2/5 [40.0%]). In the remaining cases (*n* = 5/10 [50.0%]), discontinuation of treatment according to our protocol was advised by the attending hematologist. This was due to the wake of secondary postpartum CRNMB (*n* = 3/5 [60.0%]), primary major PPH (*n* = 1/5 [20.0%]), and miscarriage in the first trimester (*n* = 1/5 [20.0%]). In 9 out of the 10 cases of treatment discontinuation, treatment was discontinued postpartum (90.0%), and the median postpartum day of discontinuation was 19.0 (IQR, 12.0-28.0).

Concerning treatment modification, 19 patients switched from their initially prescribed LMWH preparation throughout their course of treatment (*n* = 19/62; 30.6% [95% CI, 19.6%-43.7%]). Seven patients switched more than once (*n* = 7/62 [11.3%]). In total, 28 treatment modifications were recorded, with the majority occurring antepartum (*n* = 19/28 [67.9%]) and the rest occurring postpartum (*n* = 9/28 [32.1%]). Specifically, 18 patients switched from nadroparin (antepartum: *n* = 12/18 [66.7%]; postpartum: *n* = 6/18 [33.3%]), 6 patients switched from enoxaparin (antepartum: *n* = 4/6 [66.7%]; postpartum: *n* = 2/6 [33.3%]), and 4 patients switched from dalteparin (antepartum: *n* = 3/4 [75.0%]; postpartum: *n* = 1/4 [25.0%]). Of the patients who switched from nadroparin, 16 switched due to nadroparin-induced hypersensitivity skin reactions (*n* = 16/18 [88.9%]), 1 patient switched due to pain or discomfort associated with the injections (*n* = 1/18 [5.56%]), and 1 switched due to personal preferences (*n* = 1/18 [5.56%]). The reasons for switching from enoxaparin were enoxaparin-induced hypersensitivity skin reactions (*n* = 3/6 [50.0%]) and pain or discomfort when injecting LMWH (*n* = 3/6 [50.0%]). Three of the patients who switched from dalteparin did so due to injection-related pain or discomfort (*n* = 3/4 [75.0%]), and 1 did so due to dalteparin-induced hypersensitivity skin reactions (*n* = 1/4 [25.0%]). This Figure is a schematic representation of treatment discontinuation and modification under our hospital protocol in this cohort.

**Figure:**
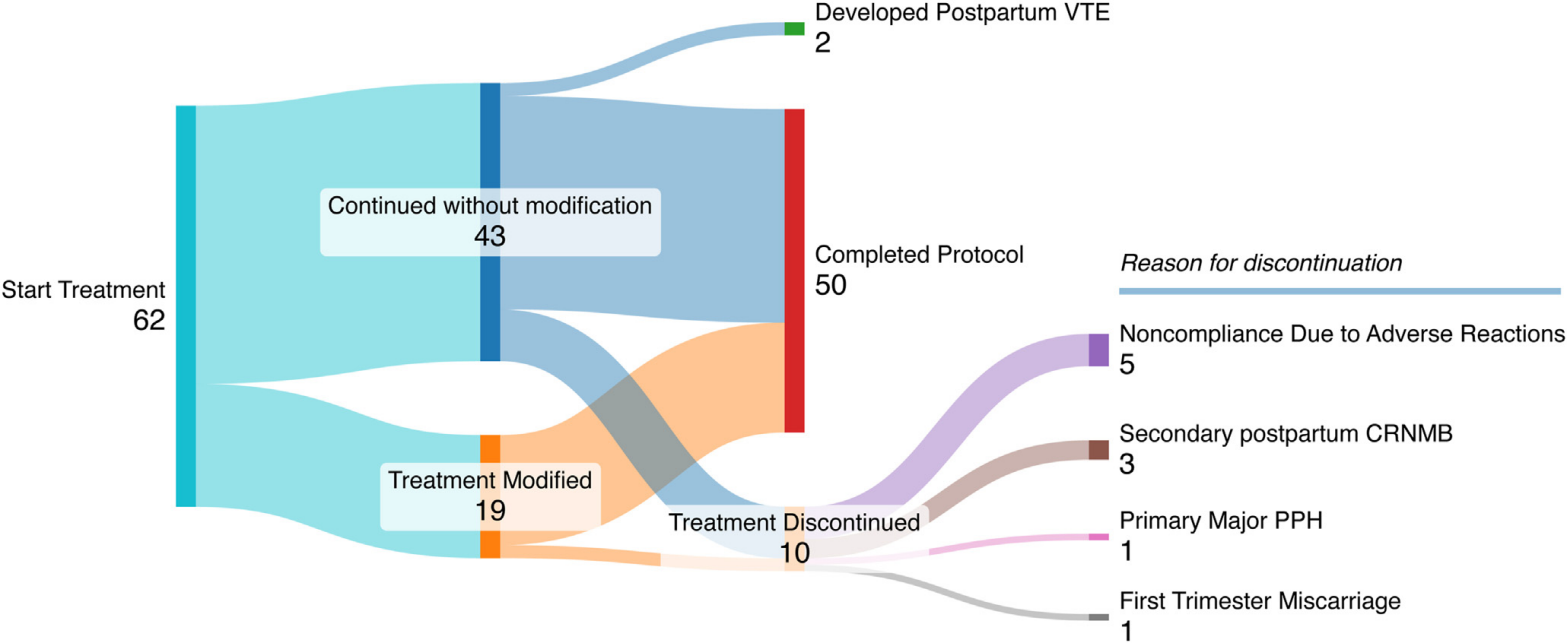
Flow diagram illustrating protocol efficacy and compliance. CRNMB, clinically relevant nonmajor bleeding; PPH, postpartum hemorrhage; VTE, venous thromboembolism.

In 10 patients, the initial administered preparation was other than our preferred LMWH—nadroparin (*n* = 10/62 [16.1%]). In most cases (*n* = 7/10 [70.0%]), this was due to a history of hypersensitivity skin reactions to nadroparin, and in the rest, the reason was not recorded (*n* = 3/10 [30.0%]). Overall, 60 patients exclusively received LMWH preparations (*n* = 60/62 [96.8%]), and 2 received fondaparinux (*n* = 2/62 [3.23%]).

## 4 Discussion

We report outcomes informing the safety and efficacy of fixed low-dose LMWH antepartum and weight-based high-dose LMWH in the puerperium in a prospective cohort of women at high risk of pregnancy-related VTE. In regard to safety, we observed a primary major PPH incidence of 6.56% (95% CI, 1.82%-15.9%), a primary postpartum CRNMB incidence of 9.84% (95% CI, 3.70%-20.2%), and a secondary postpartum CRNMB incidence of 5.00% (95% CI, 1.04%-13.9%). We did not observe any secondary major PPH. Concerning efficacy, a VTE incidence of 3.33% (95% CI, 0.406%-11.5%) was reported, with all VTEs occurring in the puerperium. Additionally, we reported on treatment discontinuation and treatment modification under our hospital protocol. We observed a high rate of treatment discontinuation (16.1% [95% CI, 8.02%-27.7%]), which was primarily postpartum, and a high rate of treatment modification (30.6% [95% CI, 19.6%-43.7%]), which was primarily antepartum.

The recently conducted Highlow study concluded that fixed low-dose LMWH is the appropriate antepartum dose of thromboprophylaxis in patients at high risk of pregnancy-related VTE but was unable to draw definite conclusions regarding the optimal postpartum dose [15]. In regard to the efficacy of weight-based high-dose LMWH in the puerperium, we found a VTE incidence in the puerperium of 3.33% (95% CI, 0.406%-11.5%). This is comparable to the incidence observed in the Highlow study in the weight-based intermediate-dose arm (1.08% [95% CI, 0.398%-2.34%]) and the fixed low-dose arm (1.98% [95% CI, 0.99%-3.52%]) [15]. Regarding the safety of weight-based high-dose LMWH in the puerperium, we did not observe any secondary major PPH (0.00% [95% CI, 0.00%-5.96%]). This is similar to the low rates of secondary major PPH observed in both arms of the Highlow study (intermediate-dose arm: 0.385% [95% CI, 0.047%-1.38%]; low-dose arm: 0.00% [95% CI, 0.00%-0.70%]) [15]. The incidence of primary postpartum CRNMB in the present study (9.84% [95% CI, 3.70%-20.2%]) was significantly higher than in the low-dose arm of the Highlow study (1.15% [95% CI, 0.425%-2.49%]) but comparable to the intermediate-dose arm (3.24% [95% CI, 1.90%-5.13%]). This is surprising, as all patients in our cohort received low-dose LMWH antepartum, and no one received high-dose LMWH prior to the recorded bleeding events. The incidence of secondary postpartum CRNMB in this study (5.00% [95% CI, 1.04%-13.9%]) was significantly higher than in both arms of the Highlow study, where no cases were observed (intermediate-dose arm: 0.00% [95% CI, 0.00%-0.70%]; low-dose arm: 0.00% [95% CI, 0.00%-0.707%]). This is an important finding, as all cases of secondary postpartum CRNMB in our study necessitated discontinuation, potentially increasing the risk of postpartum VTE for these patients.

The findings of the current study are consistent with previous cohort studies evaluating the safety and efficacy of thromboprophylaxis with various doses of LMWH in pregnancy and puerperium. A retrospective cohort study conducted by van Lennep et al. [13] reported similar rates of pregnancy-related VTE (antepartum: 1.8% [95% CI, 0.4%-9.2%]; postpartum: 7.0% [95% CI, 2.9%-16.7%]) and major PPH (9.1% [95% CI, 4.7%-16.9%]). However, van Lennep et al. [13] defined major PPH as blood loss exceeding 1000 mL, differing from the ISTH population-specific criteria employed in the current study, and they administered fixed low-dose LMWH antepartum and in the puerperium [13,19]. Galambosi et al. [20] retrospectively evaluated the outcomes of various LMWH dosing regimens in women with a history of VTE. They reported VTE incidences of 2.62% (95% CI, 1.40%-4.43%) with fixed low-dose LMWH, 5.00% (95% CI, 0.61%-16.9%) with weight-based intermediate-dose LMWH, and 0.90% (95% CI, 0.02%-4.92%) with weight-based high-dose LMWH. These findings were statistically comparable to those observed in our study [20]. Knol et al. [21], who evaluated high-dose LMWH, reported secondary PPH rates of 1.14% (95% CI, 0.029%-6.17%), which aligns with our findings. They also noted a comparable postpartum VTE rate of 0.00% (95% CI, 0.00%-5.60%). However, when comparing these findings, it is important to note that Knol et al. [21] used a different definition of PPH than the current study and administered weight-based high-dose LMWH both antepartum and in the puerperium [21].

We observed that high-dose LMWH resulted in a high incidence of premature postpartum treatment discontinuation. The causes of premature postpartum discontinuation were found to be dual-faceted: (1) due to patient noncompliance, largely attributed to adverse drug reactions, and (2) due to adverse bleeding events. In the Highlow study, there were more protocol deviations in the weight-based intermediate-dose LMWH arm than in the fixed low-dose arm [15]. Similar to observations in the current study, the protocol deviations in the Highlow study were attributed to concerns about postpartum bleeding and challenges with patient compliance [15]. Thus, there is a rationale for a lower acceptability of intermediate-dose LMWH in the High–low study compared with low-dose LMWH and poor acceptability of high-dose LMWH in the puerperium in the current study.

We observed a high rate of treatment modification in the current study. This was principally ascribed to hypersensitivity skin reactions, both antepartum and in the puerperium. The incidence of LMWH-related hypersensitivity in pregnancy and postpartum varies substantially based on the population studied [15,22,23], and very low estimates are likely a result of underreporting [24]. The incidence of LMWH-related hypersensitivity skin reactions in the current study is similar to a previous study from our research group by Schultinge et al. [25], reporting on the use of high-dose LMWH in pregnancy, and adds to the body of evidence that LMWH use during pregnancy is associated with a high rate of hypersensitivity reactions. Post hoc analysis of the findings of the Highlow trial revealed that higher doses of LMWH result in a higher incidence of hypersensitivity skin reactions (Δ = 12.7% [95% CI, 7.30%-18.1%]; *P* < .001). In the current study, we observed that adverse drug reactions, such as hypersensitivity skin reactions, affect compliance, which can reduce protocol efficacy due to treatment discontinuation. Therefore, the incidence of such adverse drug reactions is important to consider when discussing the optimal LMWH dosage strategy for thromboprophylaxis.

### 4.1 Strengths and limitations

The paucity of evidence on the optimal dosing strategy of LMWH in pregnancy and puerperium is an issue that has been voiced by major societies [6,9,11,12]. The current study is the first to report outcomes of high-risk pregnant women receiving low-dose LMWH antepartum and high-dose LMWH in the puerperium. Furthermore, the current study employs the population-specific ISTH criteria for defining bleeding events, which is specific to studies evaluating thromboprophylaxis in pregnant women [15,19]. The use of population-specific definitions of bleeding events is essential to ensure accurate estimates of the safety of LMWH.

The current study has some limitations. At our institution, we preferentially prescribe nadroparin. Therefore, caution is warranted when extrapolating our findings to other preparations. Additionally, while other preparations were used as well, our study was not designed to detect differences in outcomes when using different LMWH preparations. Furthermore, the safety outcomes of the current study are subject to information bias, as blood loss during delivery was estimated and not objectively measured. This may result in inaccurate estimates [[26], [27], [28]]. Another limitation of the current study is that we did not collect data on the ethnicity of the participants, which may limit understanding of sociocultural factors influencing the presented outcomes and the generalizability of our findings to diverse populations. An important limitation of the current study is that tracking of eligibility criteria and participant consent (including nonconsent) was not systematically recorded, and therefore, selection bias cannot be entirely excluded, which could potentially impact the generalizability of our findings. Finally, the current study is a single-center prospective cohort study, which is inherently subject to selection bias and confounding, which may further limit the generalizability of our findings.

## 5 Conclusion

In conclusion, we report a 6.56% incidence of primary major PPH, a 9.84% incidence of primary postpartum CRNMB, a 5.00% incidence of secondary postpartum CRNMB, a 3.33% incidence of postpartum VTE, a 16.1% incidence of treatment discontinuation, and a 30.6% incidence of treatment modification in 62 women with an indication for low-dose LMWH antepartum and high-dose LMWH in the puerperium. Ultimately, when evaluating the optimal dose of thromboprophylaxis, the acceptable residual risk of VTE is debatable but should be considered in conjunction with the risks of adverse events, particularly bleeding and adverse drug reactions, such as LMWH-induced hypersensitivity skin reactions.
