# Supplementary material for: High-dose postpartum thromboprophylaxis in women at high risk of pregnancy-related venous thromboembolism: a single-center prospective cohort study
Source: Res Pract Thromb Haemost. 2025 Apr 4;9(3):102846. doi: 10.1016/j.rpth.2025.102846 (PMC12861745; doi:10.1016/j.rpth.2025.102846)
Supplement: Supplementary Table S2 [file mmc2.docx]

## **S2.** Dosing of LMWH according to the UMCG protocol

| **Alternatives** | **Prophylactic doses**  (not weight-based) | **Therapeutic doses**  (weight-based) |
| --- | --- | --- |
| **First choice of the UMCG:**  Nadroparin  (Fraxiparine® or Fraxiparine Forte®) (formerly known as Fraxodi®)) | 1 dd 2.850 IE Fraxiparine® | **< 50 kg:**  2dd 3.800 IU Fraxiparine®  (with clearance 30-50 ml/min 2dd 2.850 IU)  1dd 7.600 IU Fraxiparine®  (with clearance 30-50 ml/min 1dd 5.700 IU)  **50 to 70 kg:**  2dd 5.700 IU Fraxiparine®  (with clearance 30-50 ml/min 2dd 3.800 IU)  1dd 11.400 IU Fraxiparine Forte®  (with clearance 30-50 ml/min 1dd 9.500 IU)  **70 to 90 kg:**  2dd 7.600 IU Fraxiparine®  (with clearance 30-50 ml/min 2dd 5.700 IU)  1dd 15.200 IU Fraxiparine Forte®  (with clearance 30-50 ml/min 1dd 11.400 IU)  **> 90 kg:**  2dd 9.500 IU Fraxiparine®  (with clearance 30-50 ml/min 2dd 7.600 IU)  1dd 19.000 IU Fraxiparine Forte®  (with clearance 30-50 ml/min 1dd 15.200 IU) |
| **First alternative:**  Dalteparin | 1dd 5000 IE dalteparin (Fragmin®) | **<50 kg:**  1dd 10.000 IU (with clearance 30-50 ml/min 1dd 7.500 IU, with clearance <30 1dd 5.000 IU)  **50-70 kg:**  1dd 12.500 IU (with clearance 30-50 ml/min 1dd 10.000 IU, with clearance <30 1dd 7.500 IU)  **70 to 90 kg:**  1dd 15.000 IU (with clearance 30-50 ml/min 1dd 12.500 IU, with clearance <30 1dd 7.500IE)  **90 to 110 kg:**  1dd 18.000 IU (with clearance 30-50 ml/min 1dd 15.000 IU, with clearance <30 1dd 10.000IE)  **110 to 130 kg:**  2dd 12.500 IU (with clearance 30-50 ml/min 1dd 18.000 IU, with clearance <30 1dd 12.500IE)  **130 to 150 kg:**  1dd 15.000 IU + 1dd 12.500 IU (with clearance 30-50 ml/min 2dd 10.000 IU, with clearance <30 1dd 15.000 IU)  **150 to 170 kg:**  2dd 15.000 IU (with clearance 30-50 ml/min 2dd 12.500 IU, with clearance <30 1dd 15,000IE)  **>170 kg:**  2dd 18.000 IU (for clearance 30-50 ml/min 1dd 15.000 IU + 1dd 12.500 IU, for clearance <30 1dd 18.000 IU) |
| **Second alternative:**  Enoxaparin | 1 dd 40 mg enoxaparin | **<60 kg:**  2dd 60 mg or 1 dd 100 mg enoxaparin  (for clearance 30-50 ml/min 2dd 40 or 1dd 80 mg)  **60-80 kg:**  2dd 80 mg or 1 dd 120 mg enoxaparin  (at clearance 30-50 ml/min 2dd 60 or 1dd 100 mg)  **80 to 100 kg:**  2dd 100 mg or 1 dd 160 mg enoxaparin  (for clearance 30-50 ml/min 2dd 80 or 1dd 120 mg) |

**In case of contraindication to all LMWHs:**

Fondaparinux (2.5 mg once daily subcutaneously) is used as an alternative for venous thrombosis prophylaxis when there is a contraindication to all LMWHs. A dosage of 7.5 mg once daily may be used for therapeutic anticoagulation. Unlike LMWH, fondaparinux in prophylactic dosing needs to be adjusted for renal function, to 1.5 mg with clearance of 20-50 ml/min, and it is contraindicated with clearance under 20 ml/min. There is limited experience with adjusting the therapeutic dosage of fondaparinux in impaired renal function: if clearance is less than 50 ml/min, an alternative agent is preferred. If no alternative is available, 5 mg fondaparinux may be considered with clearance of 30-50 ml/min.
